# High mortality during tuberculosis retreatment at a Ghanaian tertiary center: a retrospective cohort study

**DOI:** 10.11604/pamj.2019.33.111.18574

**Published:** 2019-06-13

**Authors:** Tara Catherine Bouton, Audrey Forson, Samuel Kudzawu, Francisca Zigah, Helen Jenkins, Tsigereda Danso Bamfo, Jane Carter, Karen Jacobson, Awewura Kwara

**Affiliations:** 1 Division of Infectious Diseases, Warren Alpert Medical School, Brown University, Providence, Rhode Island; 2 University of Ghana School of Medicine and Dentistry, Accra, Ghana; 3 Korle-Bu Teaching Hospital, Accra, Ghana; 4 Department of Biostatistics, Boston University School of Public Health, Boston, Massachusetts; 5 Division of Pulmonary, Critical Care and Sleep Medicine, Warren Alpert School of Medicine, Brown University, Providence, Rhode Island; 6 Section of Infectious Diseases, Boston University School of Medicine, Boston, Massachusetts; 7 Division of Infectious Diseases & Global Medicine, University of Florida College of Medicine, Gainesville, Florida

**Keywords:** Recurrent tuberculosis, previously treated tuberculosis, drug resistant tuberculosis

## Abstract

**Introduction:**

High mortality among individuals receiving retreatment for tuberculosis (RT-TB) persists, although reasons for these poor outcomes remain unclear.

**Methods:**

We retrospectively reviewed 394 RT-TB patients diagnosed between January 2010 and June 2016 in Accra, Ghana.

**Results:**

Of RT-TB patients, 161 (40.9%) were treated empirically (negative/absent smear, culture or Xpert), of whom 30.4% (49/161) had only extrapulmonary TB signs or symptoms. Mortality during treatment was 19.4%; 15-day mortality was 10.8%. In multivariable proportional hazards regression, living with HIV (aHR=2.69 [95 CI: 1.51, 4.80], p<0.01) and previous loss-to-follow up (aHR=8.27 (95 CI: 1.10, 62.25), p=0.04) were associated with mortality, while drug susceptibility testing (DST, aHR=0.36 (95 CI: 0.13, 1.01), p=0.052) was protective. Isoniazid resistance was observed in 40% (23/58 tested) and rifampin resistance in 19.1% (12/63 tested).

**Conclusion:**

High rates of extrapulmonary TB and smear/culture negative disease highlight the barriers to achieving DST-driven RT-TB regimens and the need for improved diagnostics. Our finding of poly-drug resistance in rifampin-susceptible cases supports access to comprehensive first line DST. Additionally, interventions to reduce mortality, especially in HIV co-infected RT-TB patients, are urgently needed.

## Introduction

To reach the WHO's End TB Strategy goal of a 90% reduction in tuberculosis (TB) deaths by 2030 [1], addressing retreatment TB (RT-TB, patients previously treated with at least one month of anti-TB drugs) is critical. RT-TB patients have both higher mortality [2] and drug-resistance rates [1] than first time TB treatment patients. From 1991 to 2017, an empiric 8-month retreatment regimen of 5 first line drugs (category II regimen) was recommended by the WHO for RT-TB, where drug susceptibility testing (DST) was unavailable or delayed [3, 4]. With increased global access to second line drugs and DST, the WHO guideline for RT-TB has moved to drug-susceptibility based regimens [4, 5]. However, DST coverage amongst bacteriologically-confirmed pulmonary RT-TB patients is less than 40% in the African region [1]. In addition, up to 65% of RT-TB patients present as smear negative, extrapulmonary or as patients without laboratory testing [6] and thus are not included in calculations of DST coverage. In this study, we assess RT-TB outcomes, rates of bacteriologic confirmation of RT-TB and factors associated with mortality in RT-TB.

## Methods

**Study population:** we performed a retrospective review of all RT-TB patients treated between January 1^st^, 2010 and May 31^st^, 2016 at Korle Bu Teaching Hospital (KBTH) Chest Clinic in Accra, Ghana. KBTH Chest Clinic serves the surrounding urban community, KBTH HIV treatment center (one of Ghana's largest), and as a referral facility for complicated adult TB cases from other regional DOT centers.

During the study period, national RT-TB guidelines mirrored WHO recommendations of empiric category II regimen (2SHRZE/1HRZE/5HRE; S-Streptomycin, H-Isoniazid, R-Rifampicin, Z-Pyrazinamide, E-Ethambutol) while awaiting DST results. Patients identified as having rifampin-resistant TB by any available result were switched to multidrug resistant TB regimen. Diagnosis of extrapulmonary TB was limited to clinical assessment and pulmonary specimens in most cases. Genotypic DST with Xpert MTB/RIF (Xpert; Cepheid, California) and MTBDR*plus* LPA assay (Hain Lifescience, Germany) were intermittently available during the study period. Acid fast bacilli smears (Ziehl Neelsen stains), mycobacterial culture (Lowenstein-Jensen media containing glycerol and pyruvate) and phenotypic DST (MGIT automated system -BD Diagnostics Systems, Maryland) were performed by the KBTH Chest laboratory. Patients were treated either as chest ward inpatients or clinic outpatients for daily injectable streptomycin during the initial two months of care, then continued monthly follow up outpatient with directly observed therapy performed by a community TB supervisor.

**Data collection:** patients identified in the medical record or register to be a RT-TB patient or treated with the category II retreatment regimen without documentation of prior treatment history were included. Chart, treatment register and lab register review was performed by a physician. Patients were additionally grouped based on their previous tuberculosis treatment outcome: relapse following cure or treatment completion; treatment failure (positive AFB smear or culture at month five or beyond); or lost to follow up (LTFU), defined as treatment gap of 2 or more consecutive months [7]. In keeping with WHO guidelines, we categorized RT-TB outcomes as treatment success (composite of cure/treatment completed), treatment failure, death, transferred out and LFTU (including unknown treatment outcome) [7].

**Statistical methods:** we compared baseline characteristics between bacteriologically-proven RT-TB patients and those empirically treated using Pearson chi-squared, Fisher's exact test and Student t tests where appropriate (Table 1). We performed univariate and multivariable cox proportional-hazard regression models for all-cause mortality (Table 2). To generate the multivariable model, we included all variables associated with mortality at p<0.2 in the univariate analysis. We considered backward selection, however all variables with p<0.2 were of interest and included in the model. The proportional hazards assumption was confirmed in the final multivariable model using weighted Schoenfeld residuals. Descriptive statistics were used to calculate distribution of drug resistance profiles based on combined genotypic and phenotypic DST for resistance to isoniazid and rifampin and on phenotypic results for ethambutol and streptomycin (Table 3). Analysis was completed using SAS 9.4. The study was approved by the University of Ghana School of Medicine and Dentistry and Lifespan Miriam Hospital institutional review boards.

**Table 1:** Retreatment tuberculosis cohort characteristics by whether diagnosis was bacteriologically proven

	Total	Bacteriologically Proven TB	Empirically Treated TB	p
Male (%)	264 (73.5%)	177 (76.0%)	87 (69.0%)	0.16
**Age, years (mean, SD)**	41.8 (13.6)	41.2 (12.8)	42.9 (14.9)	0.29
**Extrapulmonary disease (%)**	57 (14.5%)	5 (2.15%)	52* (32.3%)	<0.01**
**1 Prior TB Episodes (%)**	241 (87.0%)	138 (84.1%)	103 (91.2%)	0.09
**>1 Prior TB episodes (%)**	36 (13.0%)	26 (15.9%)	10 (8.8%)	
**HIV status (%):**				<0.01
Positive	93 (24.0%)	44 (18.9%)	49 (31.8%)	
Negative	271 (70.0%)	183 (78.5%)	88 (57.1%)	
Unknown	23 (5.9%)	6 (2.6%)	17 (11.0%)	
**Initial regimen (%):**				<0.01**
Category I	29 (7.6%)	11 (4.8%)	18 (11.6%)	
Category II	321 (84.0%)	194 (85.5%)	127 (81.9%)	
Modified category II***	24 (6.3%)	19 (8.4%)	5 (3.2%)	
Other	8 (2.1%)	3 (1.3%)	5 (3.2%)	
**Treatment outcome (%):**				<0.01
Success (cure/completed)	215 (56.4%)	135 (57.9%)	80 (54.1%)	
Failure	14 (3.7%)	14 (6%)	0 (0%)	
Death	74 (19.4%)	32 (13.7%)	42 (28.4%)	
LTFU	37 (9.7%)	26 (11.2%)	11 (7.4%)	
Transferred out	41 (10.8%)	26 (11.2%)	15 (10.1%)	
**15 Day treatment mortality** **(%)**	41 (10.8%)	17 (7.3%)	24 (16.2%)	<0.01

* Includes the 49 patients with extrapulmonary TB only (without suspicion or testing attempted for pulmonary disease), and 3 additional patients with extrapulmonary TB testing attempted for pulmonary TB

** Fisher’s Exact Test, otherwise Pearson Chi Squared or student T test

*** Modified Category II signifies use of the category II regimen without streptomycin

Abbreviations: SD, standard deviation; TB, tuberculosis; LTFU, lost to follow up

**Table 2:** Single and multivariable cox proportional-hazard regression models of mortality during retreatment

	N	Hazard ratio (95% CI) in univariate analysis	p	aHR (95% CI) in multivariable model	p
Prior treatment outcome:	238				
Failure		Referent		Referent	
LTFU		2.95 (1.01, 8.60)	0.05	8.27 (1.10, 62.25)	0.04
Relapse		2.00 (0.72, 5.61)	0.19	5.85 (0.80, 43.02)	0.08
Age at retreatment (per year older)	232	1.01 (0.99, 1.03)	0.14	1.01 (0.99, 1.03)	0.47
Male	230	1.21 (0.67, 2.19)	0.52		
More than 1 prior TB episode	193	1.14 (0.51, 2.52)	0.76		
Time since last TB treatment (per additional month)	156	1.00 (0.99, 1.01)	0.25		
Excessive alcohol use*	139	1.55 (0.54, 4.47)	0.41		
Any substance use*	139	1.70 (0.64, 4.40)	0.29		
Tobacco use*	53	0.75 (0.24, 2.38)	0.23		
HIV positive	231	2.66 (1.59, 4.44)	<0.01	2.69 (1.51, 4.80)	<0.01
On ART at TB retreatment diagnosis	39	0.82 (0.22, 3.02)	0.76		
Duration of TB symptoms (per additional months)	74	0.98 (0.91, 1.06)	0.58		
Initial sputum smear >2+	144	0.84 (0.39, 1.79)	0.65		
Cavity identified on x-ray*	102	1.27 (0.60, 2.66)	0.53		
Extrapulmonary disease	248	1.84 (1.05, 3.22)	0.03	1.78 (0.91, 3.48)	0.09
Any DST done (including Xpert only)	248	0.20 (0.07, 0.56)	<0.01	0.36 (0.13, 1.01)	0.052
Known rifampin resistance	51	1.27 (0.13, 12.71)	0.84		
Known isoniazid resistance	47	0.51 (0.04, 6.14)	0.60		
Any phenotypic resistance	41	1.04 (0.09, 11.86)	0.98		
Initiated retreatment on category 2 (vs any other Rx)	245	0.80 (0.45, 1.44)	0.46		

* Data as abstracted from clinical chart review without sufficient detail to further quantify severity

Abbreviations: CI, confidence interval; aHR, adjusted hazard ratio; LTFU, lost to follow up; DST, drug susceptibility testing; Rx, regimen

**Table 3:** Resistance patterns by drug and resistance class

Resistance:	N/available results	%
Combined geno*/phenotypic DST:		
H (including multidrug resistance)	23/58	40.0
R (including multidrug resistance)	12/63	19.1
Phenotypic DST patterns:		
HR	2/50	4.0
HRE	1/50	2.0
HRES	4/50	8.0
HRS	4/50	8.0
HE	1/50	2.0
HS	2/50	4.0
E	3/50	6.0
S	3/50	6.0
Z	Not done	
Pan-susceptible	21/50	42.0
Resistant to ≥3 of 4 tested drugs	9/50	18.0
Proportion of resistance missed if Xpert testing used alone	18/29	62.1

* Genotypic testing intermittently available during the study period for isoniazid and rifampin only

Abbreviations: DST, drug susceptibility testing; H, isoniazid; R, rifampin; E, ethambutol; S, streptomycin; Z, pyrazinamide

## Results

**Figure 1:**
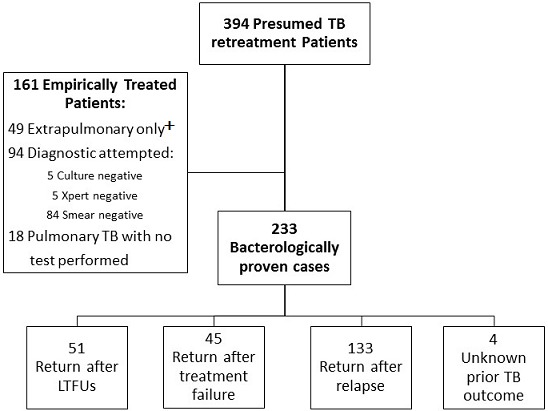
Flow diagram of presumed retreatment tuberculosis patients at Korle Bu Teaching Hospital Chest Clinic and their previous treatment outcomes. +Extrapulmonary only defined as clinical diagnosis without suspicion or testing for pulmonary disease. Abbreviations: TB, tuberculosis; LTFU, lost to follow up

**Retreatment TB patient characteristics:** of 394 total RT-TB patients, 233 (59.1%) had bacteriologically proven RT-TB (positive AFB smear, Xpert and/or mycobacterial culture), while 161 (40.9%) without bacteriologic confirmation were treated empirically based on clinical presentation consistent with RT-TB (Figure 1). Median age was 41.8 years, 73.5% were male and 24.0% were living with HIV (Table 1). Thirty percent (49/161) of empirically treated RT-TB patients had clinically diagnosed extrapulmonary TB without suspicion or testing attempted for pulmonary disease, while an additional 8 patients were diagnosed with and tested for pulmonary TB in addition to extrapulmonary TB. Of pulmonary RT-TB patients, 67.5% (233/345) had a bacterial diagnosis, 27.3% (94/345) had attempted bacterial testing (combined bacteriologically negative and uninterpretable results, eg overgrowth), and 5.2% (18/345) had no testing performed (Table 4).

**Table 4:** Bacteriologic confirmation of retreatment diagnosis amongst pulmonary retreatment tuberculosis patients

	N	%
Total pulmonary retreatment TB patients	345	
Lacked attempted bacteriologic TB confirmation	18	5.2
Unsuccessful bacteriologic TB confirmation*	94	27.3
Successful bacteriologic TB confirmation	233	67.5

* Unsuccessful bacteriologic confirmation defined as combined bacteriologically negative and uninterpretable results (overgrowth)

Abbreviations: TB, tuberculosis

Overall RT-TB treatment success was 56.4%, with mortality during treatment of 19.4% (Table 1). Thirteen percent of RT-TB patients were being treated for at least the third TB episode (Table 1). During our study window, 84% of RT-TB patients were initially started on the category II regimen. As detailed in Table 1, empirically treated RT-TB patients were more likely to initially receive the category I regimen (2HRZE/4HR, 11.6% vs 4.8%, p<0.01), while bacteriologically confirmed RT-TB patients were more likely to initially receive a modified category II regimen without streptomycin (3HRZE/5HRE, 8.4% vs 3.2%, p<0.01). Patients without bacteriologic confirmation of RT-TB were significantly more likely to be living with HIV (31.8% vs 18.9%, p<0.01), to demonstrate extrapulmonary TB (32.3% vs 2.2%, p<0.01), and had higher 15-day mortality (16.2% vs 7.3%, p<0.01), compared to those with bacteriologic confirmation (Table 1).

**Analysis of mortality:** univariate risk factors of mortality included presenting after LTFU, living with HIV, and extrapulmonary disease, while DST testing was associated with survival (Table 2). In multivariable analysis, presenting after LTFU (aHR=8.27 (95 CI: 1.10, 62.25), p=0.04) and living with HIV (aHR=2.69 (95 CI: 1.51, 4.80), p<0.01) persisted as risk factors for mortality, while completion of any DST testing (aHR=0.36 (95 CI: 0.13, 1.01), p=0.052), including Xpert alone, was associated with survival (Table 2).

**Drug resistance amongst retreatment TB patients:** forty percent (23/58) of RT-TB patients with phenotypic/genotypic DST data available were found to have isoniazid resistance, 19.1% (12/63) had rifampin resistance, 26% (13/50) had streptomycin resistance, and 18% (9/50) had ethambutol resistance (including multidrug resistance), while 42% (21/50) were susceptible to all four first line drugs tested (Table 3). Of those with phenotypic DST testing, 18% were resistant to 3 or more of the 4 first line drugs tested. Sixty two percent of resistant isolates were found to have resistance to first line drugs other than rifampin, which would have been missed had they been tested with Xpert alone (resistance to ethambutol, isoniazid and/or streptomycin) (Table 3).

## Discussion

In our cohort of RT-TB patients from a Ghanaian academic referral clinic, 19.4% of patients died during retreatment, higher than the 4-13% mortality rate of published RT-TB cohorts worldwide [8-16]. Persons returning after LTFU, living with HIV, and without DST results were at increased mortality risk.

Our findings underscore the critical-and urgent-importance of global recommendations for all RT-TB presumptive patients to have access to DST. Though availability of the empiric category II regimen during our study [4] may have decreased utilization of culture and DST, almost a third of patients did not have bacteriologic RT-TB diagnostic confirmation. Without positive smears or cultures, these patients were not able to receive DST and therefore lacked an opportunity for regimen adjustment to be considered. Consistent with previous RT-TB cohorts in Africa and Asia [6, 12, 17-19], we found significant numbers of both extrapulmonary and smear negative RT-TB patients, complicating both diagnosis and provision of DST. Implementation of molecular diagnostics, like Xpert with its increased sensitivity, increases TB detection in treatment naïve patients, but its application in RT-TB results in decreased specificity especially when used proximal to last TB treatment and identification of residual non-viable MTB DNA may occur [20]. The resulting need to utilize slower, lower sensitivity, traditional diagnostics limits RT-TB detection, results in diagnostic reliance on nonspecific clinical findings, and leads to overtreatment and missed alternative diagnoses. Reducing RT-TB mortality requires the combination of rigorous, comprehensive diagnostic investigations [21] coupled with rapid, accessible, and widely-utilized TB diagnostics, including for extrapulmonary cases.

We found that when mortality occurred, it was early in RT-TB therapy. While one study in Tanzanian RT-TB patients found 6.5% mortality in the first two months [13], we observed over 10% mortality within the first fifteen days of therapy. This high early mortality rate likely reflects late advanced disease presentations. Similarly, delayed return to care and resulting advanced disease may also explain our finding of increased mortality in those presenting after LTFU (aHR=8.27). Cohorts from India and Uganda have also shown higher mortality amongst RT-TB patients returning after LTFU [12, 14]. Attempts for early recognition of LTFU and innovated methods for rapid re-engagement in care may also be critical to lower RT-TB mortality rates.

Others have previously shown that patients with RT-TB have higher rates of drug resistance, with MDR amongst RT-TB cases estimated to be five times higher than amongst new TB cases [7, 8, 22]. Importantly, we observed additional drug-resistance patterns, including 40% isoniazid-resistance amongst tested isolates. Isoniazid resistance is associated with poor clinical outcomes [23] with recent guidelines issued for global roll out of an isoniazid-resistant regimen [5]. In our cohort, 62% of drug-resistant isolates were resistant to drugs other than rifampin (Table 3), highlighting that poly-drug resistance is missed in rifampin-susceptible cases when tested only with Xpert. Our observation that having available DST results was protective against mortality (aHR=0.36) supports universal access to first line DST to enable individualized RT-TB regimens.

Our study had several limitations influenced by its retrospective design. In this high burden TB/HIV country [1], we observed that HIV co-infection remains tied to mortality amongst RT-TB patients (aHR=2.69), however we were unable to examine the impact of late HIV diagnosis or ART on RT-TB outcome. Identification of comorbidities such as alcohol use and other substance use as well as chest x-ray findings were dependent on clinician documentation. RT-TB sub-classification often relied on patient or medical staff recall of prior treatment outcome. The inclusion of patients treated with the category II regimen without clear documentation of previous treatment history may have led to inclusion of TB treatment naïve patients in the analysis; however, they were in the minority and there were no indications for category II therapy other than retreatment. Finally, KBTH is a referral center, possibly limiting the generalizability of our findings to RT-TB patients in similar settings.

## Conclusion

Years of empiric treatment for RT-TB in low and middle resource settings, such as the application of category II regimens or blind re-application of category I, has led to inadequate treatment with resultant poor success rates, drug resistance, morbidity and mortality. With the END TB strategy’s emphasis on patient centered care and human rights [24], universal access to comprehensive first-line genotypic and phenotypic DST with individualized regimens in RT-TB is not a luxury, but an urgent imperative.

### What is known about this topic

- To curb the global tuberculosis epidemic, we must target key groups at high-risk of drug resistance and poor treatment outcomes;
- Retreatment tuberculosis patients have both higher mortality and drug-resistance rates when compared tuberculosis patients treated for the first time;
- The reasons for high mortality among retreatment tuberculosis patients are unclear.

### What this study adds

- High rates of extrapulmonary tuberculosis and smear/culture negative disease among retreatment tuberculosis patients in Ghana highlight the barriers to achieving individualized regimens and the need for improved diagnostics;
- More than half of drug-resistant isolates were resistant to drugs other than rifampin, suggesting access to comprehensive first-line drug susceptibility testing, rather than reliance on Xpert testing alone, is critical in this population;
- Early recognition of tuberculosis treatment loss to follow up, and innovated methods for rapid re-engagement in care may lower retreatment tuberculosis mortality rates.

## Competing interests

The authors declare no competing interests.
